# Predictive value of serum creatinine/cystatin C in neurocritically ill patients

**DOI:** 10.1002/brb3.1462

**Published:** 2019-11-08

**Authors:** Shengnan Wang, Ling Xie, Jiawei Xu, Yanhong Hu, Yongming Wu, Zhenzhou Lin, Suyue Pan

**Affiliations:** ^1^ Department of Neurology Nanfang Hospital Southern Medical University Guangzhou China; ^2^ Department of Neurology Hainan General Hospital Haikou China

**Keywords:** neurocritical care, poor prognosis, sarcopenia, serum creatinine to cystatin C ratio

## Abstract

**Objective:**

To explore the predictive value of serum creatinine (Cr) to cystatin C (CysC) ratio in neurocritically ill patients.

**Methods:**

We conducted a retrospectively observational study of adult patients admitted to a neurocritical care unit (NCU) between Jan 2013 and Jan 2017. Patients were excluded if <18 years old, required neurocritical care <72 hr, did operation during hospitalization, had premorbid disability or acute kidney injury (AKI) at admission. The Cr/CysC ratio was obtained at NCU admission. Primary end points were short‐term (30‐day) mortality and long‐term (6‐month) poor outcome, with the latter defined as modified Rankin Scale (mRS) of 4–6.

**Results:**

Of 538 eligible patients, the etiology included acute ischemic stroke (*N* = 193, 35.9%), intracranial hemorrhage (*N* = 116, 21.6%), encephalitis and/or meningitis (*N* = 85, 15.8%), and others (*N* = 144, 26.7%). Serum Cr/CysC ratio was significantly correlated with body mass index (BMI) (*r* = .161, *p* < .001), the length of NCU stay (*r* = −.161, *p* < .001), duration of mechanical ventilation (*r* = −.138, *p* = .001), and risk of tracheotomy (*r* = −.095, *p* = .028). During follow‐up, 88 (16.4%), patients died within 30 days and 307 (57.1%) patients achieved good outcome at 6 months. In multivariate logistic regression analysis, we identified serum Cr/CysC ratio as an independent predictor of long‐term functional outcome (OR: 0.989, 95% CI: 0.980–0.998, *p* = .015) but not 30‐day mortality (*p* = .513).

**Conclusions:**

Serum Cr/CysC ratio at admission could be used as a predictor of long‐term poor prognosis in neurocritically ill patients.

## Graphical abstract

Serum creatinine/cystatin C ratio could be used as a predictor of long‐term poor prognosis in neurocritically ill patients, and it was associated with body mass index, the length of stay in neurocritical care unit, duration of mechanical ventilation, and risk of tracheotomy as well.

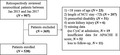

## 1 INTRODUCTION

Sarcopenia, defined as the progressive and generalized loss of skeletal muscle mass, strength, and function (performance) with a consequent risk of adverse outcomes, is often a phenomenon of the aging processes (primary sarcopenia) and also can be a result from pathogenic mechanisms (secondary sarcopenia) such as disease‐related, activity‐related (e.g., disuse), or nutrition‐related (e.g., protein deficiency) (Cederholm et al., 2017). Sarcopenia is very common in critically ill patients, with an incidence of about 60%–70% and is associated with infectious complications, prolonged duration of mechanical ventilation, longer hospitalization, greater need for rehabilitation care after hospital discharge, and higher mortality (Peterson & Braunschweig, 2016). However, in critical settings, it would be very challenging to evaluate the muscle mass.

The Sarcopenia Working Group of the European Union Geriatric Medicine Society have suggested some techniques to measure the muscle mass (Cruz‐Jentoft et al., 2010), including computed tomography (CT), magnetic resonance imaging (MRI), dual‐energy X‐ray absorptiometry (Cooper et al., 2013; Jones, Doleman, Scott, Lund, & Williams, 2015; Pagotto & Silveira, 2014), bioelectrical impedance analysis (Chien, Huang, & Wu, 2008), and total or partial body potassium per fat‐free soft tissue (Janssen, Baumgartner, Ross, Rosenberg, and Roubenoff (2004), among which CT and MRI are considered to be the golden standard. However, using such tools to evaluate muscle mass in critically ill patients is usually impractical, due to the clinical instability of the patients, the financial issues, the limited access to equipment, concerns about radiation exposure, and the requirement for consecutive monitoring.

Serum creatinine (Cr) is derived from creatine phosphate, a skeletal muscle protein. When muscle mass is stable, the production of Cr is relatively constant. Cystatin C (CysC) is a small nonionic protein excreted by all nucleated cells, and it is considered to be unaffected by any factors (e.g., muscle mass, lean tissue mass, age, ambulation, circadian rhythm, and sex) other than renal function status (Vinge, Lindergard, Nilsson‐Ehle, & Grubb, 1999). Since both the Cr and CysC are excreted from the kidney in the same manner, the Cr/CysC ratio could thereby exclude the influence of renal function and reflect the muscle mass of the body. Besides, the Cr/CysC ratio is easy to test and convenient for consecutive monitoring, and it has been reported to be associated with sarcopenia in several studies (Kashani et al., 2017; Osaka et al., 2018; Tetsuka, 2013). However, existing data are still limited and further investigation is required to validate its consistency with muscle mass and its value on outcome prediction. We conducted this study to evaluate the predictive value of serum Cr/CysC ratio on the prognosis of neurocritically ill patients.

## 2 METHODS

### 2.1 Study design and participants

We conducted a retrospectively observational study of a database of adult patients admitted to the neurocritical care unit (NCU) of Nanfang hospital, a tertiary university‐affiliated academic hospital, between Jan 2013 and Jan 2017. The criteria for NCU admission were Glasgow Coma Scale (GCS) <12 and/or admission Acute Physiology and Chronic Health Evaluation (APACHE) II score >15; otherwise they were still included if meeting one of the criteria as reported previously (Su et al., 2009). Patients were excluded if they were younger than 18 years old, required neurocritical care for <72 hr, did operation during hospitalization, had premorbid disability (with modified Rankin Scale (mRS) of > 1), or acute kidney injury (AKI) (Kellum & Lameire, 2013) at admission. We excluded the patients with AKI since at that situation serum Cr and CysC have variable kinetics and the Cr/CysC ratio may no longer indicate the muscle mass precisely (Kashani et al., 2017). Patients with missing data (no CysC at admission, insufficient data for APACHE II score, or loss to follow‐up) and data of rehospitalization were also excluded. The study proposal was approved by the Nanfang hospital's ethics committee for clinical research. Informed consent was waived by the review board because this study was retrospective, observational, and all data were fully de‐identified.

### 2.2 Study variables

Electronic medical records were carefully reviewed to collect the patient information of demographics, diagnoses, medical history, GCS scores, APACHE II scores, laboratory values, length of NCU stay, duration of mechanical ventilation, incidence of intubation, and tracheotomy. The Cr/CysC ratio was calculated from serum Cr and CysC both obtained at the time of NCU admission. GCS scores were extracted from the first neurological examination at NCU admission. The total scores of APACHE II (Vincent et al., 1996) were obtained according to the corresponding parameters within the first 24 hr of NCU admission.

### 2.3 Study outcomes

Primary end points were short‐term (30‐day) mortality and long‐term (6‐month) poor outcome, with the latter defined as mRS of 4–6. Information on survival and functional status was obtained through telephone interview by a trained neurologist blinded to the study data.

### 2.4 Statistical analysis

All analyses were performed using SPSS, version 20.0 (SPSS). Categorical variables were presented as number (%), and continuous data were presented as mean ± standard deviation (*SD*) or median (25%–75% interquartile range [IQR]), as appropriate. Correlations between variables were determined with the Spearman's Rank Correlation test. To evaluate the performance of sarcopenia index at admission in predicting 30‐day mortality and 6‐month poor outcome, we used multivariate logistic regression model adjusted for age, gender, etiology, APACHE II score, and duration of mechanical ventilation. The 95% confident intervals (CIs) reported for the logistic regression odds ratios (ORs) were calculated by the maximum likelihood estimation (forward selection). A two‐sided *p* value <.05 was considered to be statistically significant.

## 3 RESULTS

**Figure 1:**
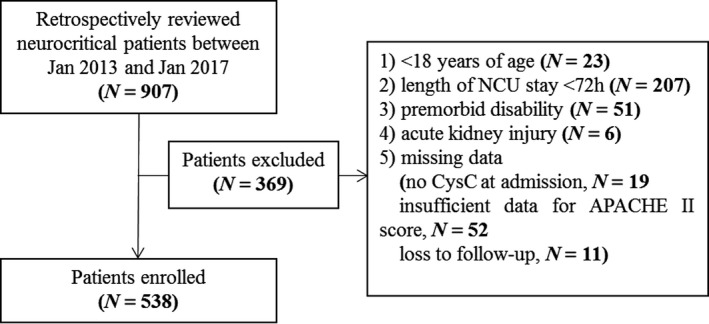
Patient inclusion flowchart. APACHE, Acute Physiology and Chronic Health Evaluation; CysC, cystatin C; NCU, neurocritical care unit

**Figure 2:**
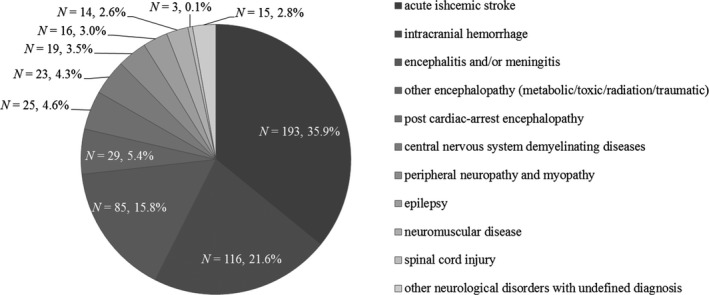
Categories of etiology

Of 907 patients screened for eligibility, 538 satisfied inclusion and exclusion criteria (Figure 1). All the patients were admitted for primary neurological disorders, with etiology of acute ischemic stroke (*N* = 193, 35.9%), intracranial hemorrhage (*N* = 116, 21.6%), encephalitis and/or meningitis (*N* = 85, 15.8%), encephalopathy (metabolic/toxic/radiation/traumatic) (*N* = 29, 5.4%), post‐cardiac arrest encephalopathy (*N* = 25, 4.6%), central nervous system demyelinating diseases (*N* = 23, 4.3%), peripheral neuropathy and myopathy (*N* = 19, 3.5%), epilepsy (*N* = 16, 3.0%), neuromuscular disease (*N* = 14, 2.6%), spinal cord injury (*N* = 3, 0.1%), and other neurologic disorders with undefined diagnosis (*N* = 15, 2.8%) (Figure 2). In this cohort, the median (IQR) age was 57 (44–69) years and 340 (63.2%) were male. The median (IQR) GCS was 11 (7–12), and APACHE II score was 15 (10–20). The median (IQR) onset‐to‐admission duration was 5 (1–14) days, and the mean body mass index (BMI) was 22.9 ± 2.9 kg/m^2^. The mechanical ventilation, intubation, and tracheotomy ratio were 33.6%, 50.9%, and 30.1%, respectively. The median (IQR) of admission serum creatinine, cystatin C, and Cr/CysC ratio were 71 (53–91) mmol/L, 1.02 (0.81–1.31) mg/L, and 69.84 (55.31–85.43), respectively. Other baseline demographics and clinical characteristics of the patients were summarized in Table 1.

**Table 1:** Characteristics of enrolled patients

Variables	*N* = 538
Demographics	
Age (years, median, IQR)	56.5 (44.0, 69.0)
Male (*n*, %)	340 (63.2)
Body mass index (kg/m^2^, mean ± *SD*)	22.9 ± 2.9
Onset‐to‐admission (days, median, IQR)	5.0 (1.0,14.0)
Diagnosis (*n*, %)	
Acute ischemic stroke	193 (35.9)
Intracranial hemorrhage	116 (21.6)
Encephalitis and/or meningitis	85 (15.8)
Neuromuscular disease	14 (2.6)
Other neurologic	130 (24.1)
Chronic conditions	
Hypertension (*n*, %)	249 (46.3)
Diabetes mellitus (*n*, %)	92 (17.1)
Critical indicators on NCU admission	
GCS (median, IQR)	11.0 (7.0, 12.0)
APACHE II score (median, IQR)	15.0 (10.0, 20.0)
Laboratory indicators	
Albumin (g/L, mean ± *SD*)	37.23 ± 5.92
Creatinine (μmol/L, median, IQR)	71.0 (53.0, 91.0)
Cystatin C (mg/L, median, IQR)	1.02 (0.81, 1.31)
Cr/CysC ratio (median, IQR)	69.84 (55.31, 85.43)
Length of stay in NCU (days, median, IQR)	10.0 (6.0, 18.0)
Mechanical ventilation (*n*, %)	181 (33.6)
Duration of mechanical ventilation (days, median, IQR)	0.0 (0.0, 4.0)
Intubation (*n*, %)	274 (50.9)
Tracheotomy (*n*, %)	162 (30.1)

Abbreviations: APACHE, Acute Physiology and Chronic Health Evaluation; Cr, creatinine; CysC, cystatin C; GCS, Glasgow Coma Scale; IQR, interquartile range; NCU, neurocritical care unit; *SD*, standard deviation.

The correlation analysis showed that the Cr/CysC ratio at admission significantly correlated with BMI (*r* = .161, *p* < .001), the length of NCU stay (*r* = −.161, *p* < .001), duration of mechanical ventilation (*r* = −.138, *p* = .001), and risk of tracheotomy (*r* = −.095, *p* = .028) (Table 2).

**Table 2:** Correlations between serum Cr/CysC ratio at admission and selected variables

Variables	*r*	*p*
Body mass index	.161	<.001
Length of NCU stay	−.161	<.001
Duration of mechanical ventilation	−.138	.001
Intubation	−.081	.061
Tracheotomy	−.095	.028

Abbreviations: Cr, creatinine; CysC, cystatin C; NCU, neurocritical care unit.

During follow‐up, 88 (16.4%) patients died within 30 days after admission and 307 (57.1%) patients achieved good outcome at 6 months. In the multivariate models evaluating the Cr/CysC ratio in outcome prediction of neurocritically ill patients, we found that the Cr/CysC ratio was significantly associated with 6‐month poor outcome (OR: 0.989, 95% CI: 0.980–0.998, *p* = .015) but not 30‐day mortality (*p* = .513), when adjusted for age, gender, etiology, APACHE II score, and mechanical ventilation (Table 3).

**Table 3:** Multivariate logistic regression analysis for serum Cr/CysC ratio on 30‐day mortality and 6‐month poor outcome

Variables	30‐day mortality		6‐month poor outcome	
	OR (95% CI)	*p*	OR (95% CI)	*p*
APACHE II score	1.281 (1.200–1.368)	<.001	1.238 (1.187–1.291)	<.001
Cr/CysC ratio	–	.513	0.989 (0.980–0.998)	.015
Mechanical ventilation	2.207 (1.173–4.149)	.014	–	.685

Note

These factors were also adjusted in the multivariate regression analysis: age, gender, etiology.

Abbreviations: APACHE, Acute Physiology and Chronic Health Evaluation; Cr, creatinine; CysC, cystatin C.

## 4 DISCUSSION

In this study, we verified serum Cr/CysC ratio as a predictor of clinical outcome in a cohort of neurocritically ill patients, and found that the Cr/CysC ratio was independently associated with 6‐month functional prognosis but not 30‐day mortality.

Because of the different origination but similar excretion pathway of Cr and CysC, the Cr/CysC ratio remains almost constant despite the renal function and could be theoretically considered to be a fair surrogate marker of muscle mass. Kashani and colleagues (Kashani et al., 2017) defined the Cr/CysC ratio as sarcopenia index and compared it with the quantification of the paraspinal muscle surface area at the 4th lumbar vertebrae by an abdominal CT scan in 226 adult critically ill patients. They found the correlation between sarcopenia index and muscle mass is 0.62 and coefficient of determination is 0.27. In another study of 62 patients with amyotrophic lateral sclerosis, the Cr/CysC ratio was shown to be associated with the severity of muscle loss (Tetsuka, 2013). Recently, a study in 285 patients with type 2 diabetes also indicated that the Cr/CysC ratio was consistent with the skeletal muscle mass estimated by bioelectrical impedance and could be used as a practical screening marker for sarcopenia (Osaka et al., 2018). In our study, we did not use the above methods except the Cr/CysC ratio to evaluate the muscle mass, due to the clinical instability of the patients at NCU admission. Nevertheless, we confirmed the Cr/CysC ratio as an independent predictor of the long‐term prognosis of neurocritically ill patients, which should be attributed to its consistency with sarcopenia.

Sarcopenia has been widely reported to be associated with more infectious complications (Lieffers, Bathe, Fassbender, Winget, & Baracos, 2012), prolonged mechanical ventilation (Moisey et al., 2013), higher mortality (Hara et al., 2016; Kashani et al., 2017; Moisey et al., 2013), longer hospitalization (Gariballa & Alessa, 2013), readmission to the hospital (Gariballa & Alessa, 2013), and greater need for rehabilitation care after hospital discharge (Lieffers et al., 2012) in diverse clinical settings. However, its importance for critically ill patients, not only the elderly, is insufficient. Here, we assessed the value of the Cr/CysC ratio, a surrogate marker for sarcopenia, in a neurocritically ill patient group of all ages. Consistent with former studies, we found that the Cr/CysC ratio was associated with the length of NCU stay, duration of mechanical ventilation, and risk of tracheotomy. Moreover, in multivariate logistic regression analysis, the Cr/CysC ratio was independently associated with long‐term functional outcome, after adjusted for confounding factors (etiology, age, gender, APACHE II score, mechanical ventilation). The result could be explained by the correlation between sarcopenia and frailty and behavioral disorders. Furthermore, a recent systemic review demonstrates sarcopenia is independently associated with cognitive impairment, which disturbs the independence of the patients (Chang, Hsu, Wu, Huang, & Han, 2016). Unlike other studies (Ju et al., 2018; Kashani et al., 2017; Moisey et al., 2013; Toptas et al., 2018), we did not find significant association between the Cr/CysC ratio and short‐term mortality. This result was perhaps due to the specificity of neurocritical illness, which per se has certain mortality, minimizing the impact of sarcopenia. For example, in this study, the most common etiology was stroke (57.5%), the mortality of which in South China is reported to be approximately 42% (Wang et al., 2017).

There are several limitations about this study. First, the retrospective nature of this study makes it susceptible to selection and information bias. Second, we were unable to assess all potentially relevant variables. Third, we did not evaluate the consistence between the Cr/CysC ratio and muscle mass due to the clinical instability of the neurocritically ill patients. Further studies should be done to validate the correlation between the Cr/CysC ratio and sarcopenia, and the impact of treatment strategies for sarcopenia on the prognosis is promising.

## 5 CONCLUSION

Serum Cr/CysC ratio could be used as a predictor of long‐term poor prognosis in neurocritically ill patients, and it was associated with the length of NCU stay, duration of mechanical ventilation, and risk of tracheotomy as well. Further studies were required to validate the correlation between the Cr/CysC ratio and sarcopenia, and the impact of treatment strategies for sarcopenia on the prognosis should be promising.

## CONFLICT OF INTEREST

None declared.

## Data Availability

The data that support the findings of this study are available on request from the corresponding author. The data are not publicly available due to privacy or ethical restrictions.
